# Neonatal Hypocalcemic Seizures in Offspring of a Mother With Familial Hypocalciuric Hypercalcemia Type 1 (FHH1)

**DOI:** 10.1210/clinem/dgaa111

**Published:** 2020-03-09

**Authors:** Poonam Dharmaraj, Caroline M Gorvin, Astha Soni, Nick D Nelhans, Mie K Olesen, Hannah Boon, Treena Cranston, Rajesh V Thakker, Fadil M Hannan

**Affiliations:** 1 Department of Paediatric Endocrinology, Alder Hey Children’s NHS Foundation Trust, Liverpool, UK; 2 Academic Endocrine Unit, Radcliffe Department of Medicine, University of Oxford, Oxford, UK; 3 Department of Paediatrics, Wrexham Maelor Hospital, Wrexham, UK; 4 Oxford Molecular Genetics Laboratory, Churchill Hospital, Oxford, UK; 5 Nuffield Department of Women’s and Reproductive Health, University of Oxford, Oxford, UK

**Keywords:** hypercalcemia, hypocalcemia, hypoparathyroidism, calcium-sensing receptor, mutation, loss-of-function

## Abstract

**Context:**

Familial hypocalciuric hypercalcemia type 1 (FHH1) is caused by loss-of-function mutations of the calcium-sensing receptor (CaSR) and is considered a benign condition associated with mild-to-moderate hypercalcemia. However, the children of parents with FHH1 can develop a variety of disorders of calcium homeostasis in infancy.

**Objective:**

The objective of this work is to characterize the range of calcitropic phenotypes in the children of a mother with FHH1.

**Methods:**

A 3-generation FHH kindred was assessed by clinical, biochemical, and mutational analysis following informed consent.

**Results:**

The FHH kindred comprised a hypercalcemic man and his daughter who had hypercalcemia and hypocalciuria, and her 4 children, 2 of whom had asymptomatic hypercalcemia, 1 was normocalcemic, and 1 suffered from transient neonatal hypocalcemia and seizures. The hypocalcemic infant had a serum calcium of 1.57 mmol/L (6.28 mg/dL); normal, 2.0 to 2.8 mmol/L (8.0-11.2 mg/dL) and parathyroid hormone of 2.2 pmol/L; normal 1.0 to 9.3 pmol/L, and required treatment with intravenous calcium gluconate infusions. A novel heterozygous p.Ser448Pro CaSR variant was identified in the hypercalcemic individuals, but not the children with hypocalcemia or normocalcemia. Three-dimensional modeling predicted the p.Ser448Pro variant to disrupt a hydrogen bond interaction within the CaSR extracellular domain. The variant Pro448 CaSR, when expressed in HEK293 cells, significantly impaired CaSR-mediated intracellular calcium mobilization and mitogen-activated protein kinase responses following stimulation with extracellular calcium, thereby demonstrating it to represent a loss-of-function mutation.

**Conclusions:**

Thus, children of a mother with FHH1 can develop hypercalcemia or transient neonatal hypocalcemia, depending on the underlying inherited CaSR mutation, and require investigations for serum calcium and CaSR mutations in early childhood.

Familial hypocalciuric hypercalcemia type 1 (FHH1) is an autosomal dominant disorder caused by loss-of-function mutations of the calcium-sensing receptor (CaSR), which is encoded by the *CASR* gene on chromosome 3q21.1 (1). The CaSR regulates calcium homeostasis by enhancing intracellular calcium (Ca^2+^_i_) mobilization and stimulating the mitogen-activated protein kinase (MAPK) cascade (2), which in turn reduces parathyroid hormone (PTH) secretion and increases urinary calcium excretion. FHH1 is generally benign and characterized by mild-to-moderate elevations of serum calcium, normal or mildly raised serum PTH, and a calcium-to-creatinine clearance ratio of less than 0.01 (1). However, the offspring of individuals with FHH1 may manifest a variety of disturbances of calcium homeostasis (3). Thus, children inheriting biallelic loss-of-function CaSR mutations from FHH1 parents can develop neonatal severe primary hyperparathyroidism (4). In addition, children inheriting a monoallelic loss-of-function CaSR mutation from the father are at risk of neonatal severe primary hyperparathyroidism if the mother is normocalcemic, whereas they would likely develop the more benign phenotype of FHH1 if they inherited the loss-of-function CaSR mutation from the mother (5), and can occasionally be normocalcemic because of incomplete penetrance of the CaSR mutation (6). Furthermore, maternal hypercalcemia caused by FHH1 may potentially cause transient hypoparathyroidism in the unaffected offspring (3), and such hypocalcemia has been reported in 2 unrelated neonates born to mothers with clinically diagnosed FHH (7,8), although the *CASR* mutation status in these cases was not established. Here, we report a 3-generation FHH1 kindred with a novel loss-of-function *CASR* mutation (p.Ser448Pro), in which an affected mother, who was heterozygous for the CaSR mutation, had 4 children, 2 of whom had asymptomatic hypercalcemia in association with the heterozygous CaSR Ser448Pro mutation, 1 without a CaSR mutation was normocalcemic, whereas another, also without a CaSR mutation, had hypocalcemic seizures as a consequence of transient neonatal hypoparathyroidism.

## Participants and Methods

### Participants

**Figure 1.**
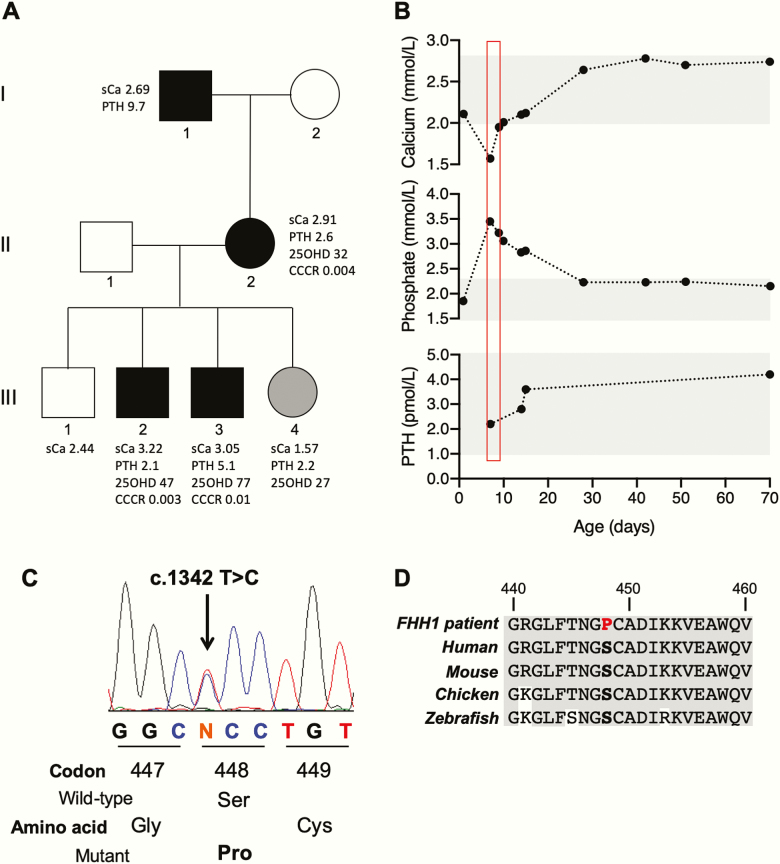
A, Pedigree and biochemistry of the family with familial hypocalciuric hypercalcemia type 1 (FHH1). Male and female family members are represented by squares and circles, respectively. Affected and unaffected individuals are represented by filled and open symbols, respectively. The hypocalcemic infant is shown in gray. B, Serum concentrations of adjusted-calcium, phosphate, and parathyroid hormone (PTH) in the hypocalcemic infant during the first 70 days postpartum. The red box indicates period of treatment with intravenous calcium and magnesium. Normal ranges are indicated by the gray areas. C, A heterozygous T > C transition at nucleotide c.1342 was identified in the hypercalcemic family members, which changes a TCC codon to CCC and is predicted to result in a missense amino acid substitution from Ser to Pro at position 448 in the CaSR protein. D, Multiple protein sequence alignment showing evolutionarily conservation of the calcium-sensing receptor (CaSR) Ser448 residue (bold). The gray area indicates conserved CaSR residues. The mutant (m) Pro448 residue is shown in red. 25OHD, 25-hydroxyvitamin D; CCCR, calcium-to-creatinine clearance ratio; sCa, serum calcium.

The three generation FHH1 kindred comprised a man (individual I.1, Fig. 1A) with hypercalcemia and his daughter (individual II.2), who had hypercalcemia and hypocalciuria, and her 4 children (individuals III.1-4). Of the 4 children, 2 (individuals III.2 and III.3) had asymptomatic hypercalcemia, 1 was normocalcemic (individual III.1), and 1 (individual III.4) developed neonatal hypocalcemic seizures. Individual III.4, a girl, was born at 38 weeks’ gestation following an uncomplicated pregnancy to nonconsanguinous parents of white European origin. She was well and normocalcemic at birth (serum adjusted-calcium 2.11 mmol/L [8.44 mg/dL]; normal, 2.00-2.80 mmol/L [8.0–11.2 mg/dL]) but was readmitted to the hospital at age 7 days with focal seizures affecting her upper limbs. Investigations showed a low serum adjusted-calcium of 1.57 mmol/L (6.28 mg/dL), elevated serum phosphate of 3.45 mmol/L (10.7 mg/dL); normal, 1.30 to 2.60 mmol/L (4.0-8.0 mg/dL), and an inappropriately normal serum PTH of 2.2 pmol/L; normal, 1.0 to 9.3 pmol/L (Fig. 1A and 1B). Serum magnesium was borderline low at 0.62 mmol/L (1.5 mg/dL); normal, 0.62 to 1.00 mmol/L (1.5-2.4 mg/dL), and serum 25-hydroxyvitamin D was low at 27 nmol/L; adequate is greater than 50 nmol/L. These findings indicated that she had hypoparathyroidism and vitamin D deficiency. She did not have clinical or biochemical features of the Di George or hypoparathyroidism-deafness-renal anomalies syndromes (9). She was treated with intravenous infusions of 10% calcium gluconate (0.5 mL/kg over 30 minutes), magnesium sulfate (100 mg/kg over 10 minutes), phenobarbitone (5 mg/kg on 3 consecutive days), and also given cholecalciferol (1000 IU daily). Over the next 48 hours her serum calcium concentration increased into the normal range (Fig. 1B). This rapid improvement in serum calcium suggested a transient cause for the hypoparathyroidism, most likely secondary to the maternal hypercalcemia. She was discharged at age 3 weeks with normal serum calcium (Fig. 1B) and no further symptoms.

### Mutational analysis

Analysis of the *CASR* gene was performed using leukocyte DNA following informed consent. Sanger sequencing of all coding exons and exon-intron boundaries was undertaken with exon-specific primers (Sigma Aldrich), and using the Big Dye Terminator v3.1 Cycle Sequencing Kit (Life Technologies), and an automated detection system (ABI3730 Automated capillary sequencer; Applied Biosystems), as reported (10, 11). Investigation of potentially pathogenic variants was undertaken using the publicly accessible Genome Aggregation Database (gnomAD) database: (https://gnomad.broadinstitute.org/), which is a dataset comprising 125 748 exome sequences and 15 708 whole-genome sequences from unrelated individuals.

### Protein sequence alignment and structural analysis

Protein sequences of CaSR orthologs were aligned using Clustal Omega (http://www.ebi.ac.uk/Tools/msa/clustalo/) (12). CaSR 3-dimensional modeling was undertaken using a reported crystal structure of the CaSR extracellular domain (ECD) (Protein Data Bank accession No. 5K5S) (13). Molecular modeling was performed using the PyMOL Molecular Graphics System (version 1.2r3pre, Schrödinger, LL Pymol) (10).

### Cell culture and transfection

Studies were performed in HEK293 cells maintained in DMEM-Glutamax media (Thermo Fisher) with 10% fetal bovine serum (Gibco) at 37ºC, 5% CO_2_. Mutations were introduced into a construct expressing WT CaSR tagged with enhanced green fluorescent protein (pEGFP-N1-CaSR) by site-directed mutagenesis using the Quikchange Lightning Kit (Agilent Technologies) and gene-specific primers (Sigma Aldrich), as described (14). WT and mutant pEGFP-N1-CaSR constructs, and luciferase reporter constructs (pGL4.30–nuclear factor of activated T cells (NFAT) and pGL4.33–serum-response element [SRE], Promega) were transiently transfected into HEK293 cells using Lipofectamine 2000 (Life Technologies) 48 hours before the experiments, as described (14, 15). Successful transfection was confirmed by visualizing green fluorescent protein (GFP) fluorescence using an Eclipse E400 fluorescence microscope with an epifluorescence filter, and images were captured using a DXM1200C digital camera and NIS Elements software (Nikon) (11, 14, 15).

### Western blot analyses

Expression of WT and mutant proteins by the pEGFP-N1-CaSR constructs was assessed by Western blot analysis, with the calnexin housekeeping protein being used as a loading control. For blots under reducing conditions, lysates were mixed with Laemmli loading buffer containing β-mercaptoethanol (BioRad) and run in Tris-Glycine–sodium dodecyl sulfate buffer. For blots under nonreducing (native) conditions, lysates were mixed with Laemmli buffer without β-mercaptoethanol and run in Tris-Glycine buffer. The plasma membrane protein fraction was isolated from cells transfected with pEGFP-N1-CaSR constructs using a plasma membrane extraction kit (Abcam, catalog No. 65400), as described (16). Plasma membrane calcium ATPase (PMCA1) protein was used as a loading control for plasma membrane fractions (16). The following primary antibodies were used for Western blot analysis: anti-CaSR (ADD, ab19347, Abcam), anti-calnexin (MA3-027, Thermo Fisher Scientific) and anti-PMCA1 (ab190355, Abcam). The Western blots were visualized using an Immuno-Star WesternC kit (BioRad) on a BioRad Chemidoc XRS+ system (11, 14, 15). Densitometry was performed using ImageJ (National Institutes of Health) and analyzed using GraphPad Prism. CaSR protein abundance was normalized to the protein loading control and expressed relative to WT CaSR. Statistical analysis was performed using the student t test.

### Functional assays

Luciferase reporter assays were undertaken to measure CaSR-mediated SRE and NFAT responses, as previously described (14, 15). Cells were transiently transfected with 100 ng/ml of the WT or mutant pEGFP-N1-CaSR constructs, 100 ng/ml luciferase construct (either pGL4-NFAT or pGL4-SRE) and 10 ng/ml pRL null control luciferase reporter. At 48 hours post-transfection, cells were then treated with 0.1 to 10 mM CaCl_2_ and incubated for 4 hours before lysis and measurement of luciferase activity using Dual-Glo Luciferase (Promega) on a Veritas Luminometer (Promega), as previously described (14). Luciferase to renilla ratios were expressed as fold-changes relative to responses at basal CaCl_2_ concentrations (0.1 mM). All assay conditions were performed in 4 to 8 biological replicates. Nonlinear regression of the concentration-response curves was performed with GraphPad Prism (GraphPad) to calculate the half-maximal (EC_50_) and maximal (E_max_) responses for each separate experiment. Statistical analysis was performed by 2-way ANOVA with Tukey multiple-comparisons test, and significant alterations in EC_50_ values were assessed using the *F* test (17).

## Results

**Figure 2.**
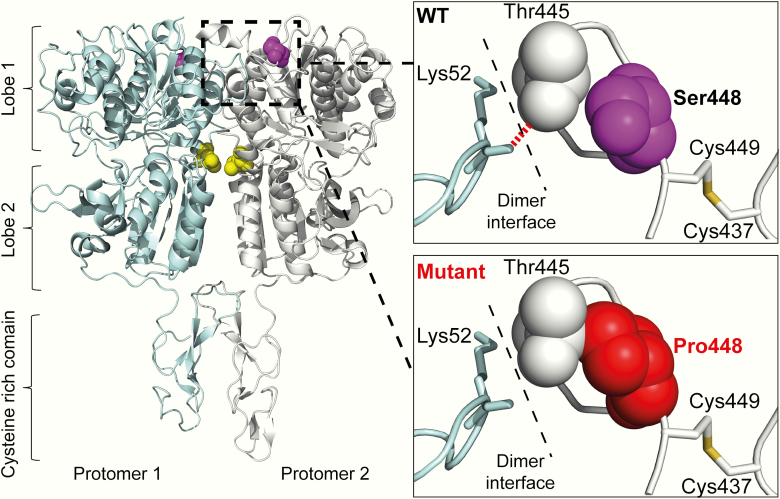
Ribbon diagram of the dimeric calcium-sensing receptor (CaSR) extracellular domain (ECD), which is derived from a published crystal structure (13). The CaSR ECD comprises a bilobed venus-fly-trap domain and a cysteine-rich domain (CRD). The WT Ser448 residue (purple) is located in lobe 1 of the CaSR ECD. The Leu173 residue, which is the site of a reported familial hypocalciuric hypercalcemia type 1 (FHH1)-causing Leu173Pro mutation (17), is shown in yellow. A close-up view shows the wild-type (WT) Ser448 residue (purple) to be in close proximity to a Thr445 residue, which is located at the extracellular dimer interface. The Thr445 residue forms a hydrogen bond (red dashed line) with the Lys52 residue of the neighboring CaSR protomer. The introduction of a mutant Pro448 residue (red) is predicted to sterically interfere with the Thr445 residue, thereby disrupting the Thr445-Lys52 interaction. The WT Ser448 residue is also located near a Cys449-Cys437 disulfide bond (yellow), and the p.Ser448Pro mutation may disrupt this interaction.

**Figure 3.**
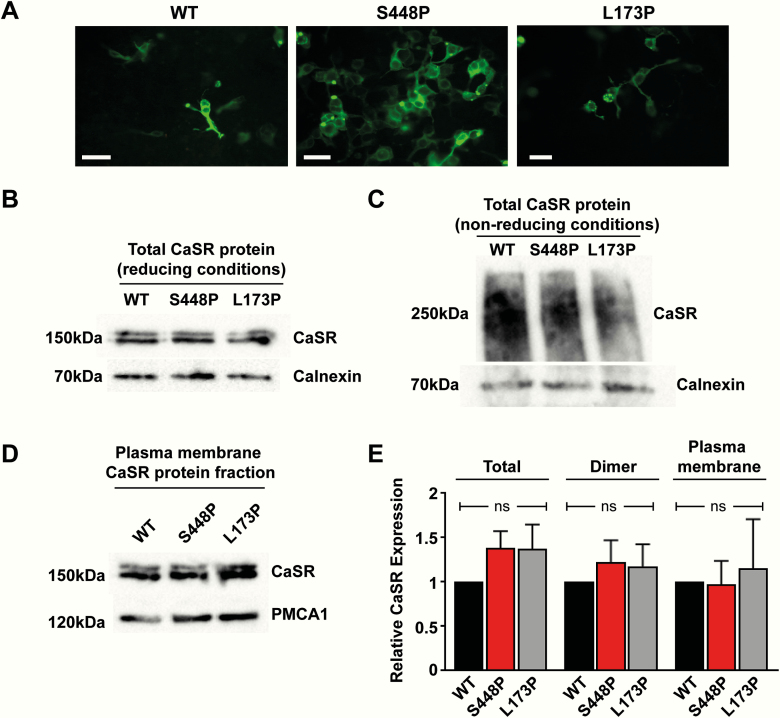
A, Fluorescence microscopy of HEK293 cells transiently transfected with pEGFP-N1–calcium-sensing receptor (CaSR) constructs expressing wild-type (WT) (Ser448) or mutant (m) (Pro448) CaSR proteins, or a known familial hypocalciuric hypercalcemia type 1 (FHH1)-causing (Leu173Pro) mutant CaSR protein. Green fluorescent protein expression in these cells indicates successful transfection and expression by these constructs. Bar indicates 10 μm. B to D, Western blot analysis of cells transiently transfected with WT or mutant CaSR proteins. B, Analysis of whole-cell lysates under reducing conditions. Calnexin was used as a loading control and the blot shown is representative of n = 5 independent experiments. C, Analysis of whole-cell lysates under nonreducing conditions to detect dimeric CaSR. Calnexin was used as a loading control and the blot shown is representative of n = 3 independent experiments. D, Western blot analysis of plasma membrane fractions. Plasma membrane calcium ATPase (PMCA1) was used as a loading control and the blot shown is representative of n = 6 independent experiments. E, Densitometric analysis of relative CaSR abundance shown as mean ± SEM. NS, nonsignificant.

DNA sequence analysis identified a novel germline heterozygous *CASR* variant (c.1342 T > C; p.Ser448Pro) in the hypercalcemic children, mother, and grandfather (Fig. 1C). However, this *CASR* variant was absent in the hypocalcemic infant and her normocalcemic sibling. The p.Ser448Pro variant was shown to affect an evolutionarily conserved residue (Fig. 1D), and this variant was not detected in the gnomAD database. The WT Ser448 residue is located in the CaSR ECD (Fig. 2). The structural consequences of the p.Ser448Pro variant were assessed using a reported crystal structure of the dimeric CaSR ECD (Fig. 2) (13). The introduction of the variant Pro448 CaSR residue was predicted to disrupt a hydrogen bond interaction across the CaSR dimer interface involving the CaSR ECD Thr445 and Lys52 residues, and also potentially affect a disulfide bond formed between the neighboring Cys449 and Cys437 residues (Fig. 2). To determine the effects of these predicted structural changes on CaSR-mediated signaling, HEK293 cells were transiently transfected with pEGFP-N1-CaSR constructs expressing WT (Ser448) or variant (Pro448) CaSR proteins. A reported FHH1-causing (Leu173Pro) CaSR protein (Fig. 3) was used as a control loss-of-function mutation (17). CaSR expression was confirmed by fluorescence microscopy and Western blot analysis (Fig. 3A and 3B). Western blot analysis was also performed under nonreducing conditions to detect dimeric CaSR proteins, and on plasma membrane protein fractions of cells transiently transfected with pEGFP-N1-CaSR constructs (Fig. 3C and 3D). Significant alterations were not detected in the expression of total cellular, dimeric, or plasma membrane forms of the Pro448 and Pro173 CaSR proteins compared to WT CaSR (Fig. 3E).

**Figure 4.**
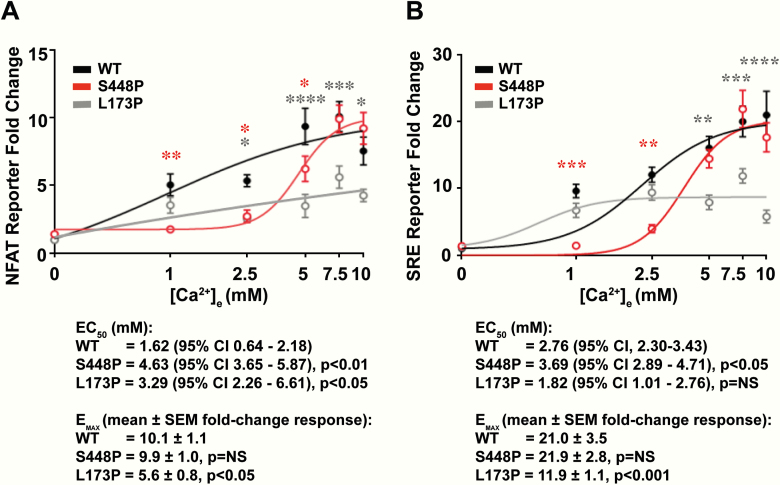
Concentration-response curves of extracellular calcium (Ca^2+^_e_)-induced: A, nuclear factor of activated T-cells (NFAT) and B, serum-response element (SRE) luciferase reporter responses of HEK293 cells expressing wild-type (WT) (black) or mutant (Pro448, red) calcium-sensing receptor (CaSR) proteins, or a known familial hypocalciuric hypercalcemia type 1 (FHH1)-causing (Leu173Pro, gray) CaSR mutant protein. Responses at each Ca^2+^_e_ concentration are expressed as a fold-change of basal [Ca^2+^]_e_ responses and shown as mean ± SEM of 4 to 8 biological replicates. The half-maximal (EC_50_) and maximal fold-change values are shown below the concentration-response curves. NS, nonsignificant, ****P less than .0001, ***P less than .001, **P less than .01, *P less than .05 compared to WT.

The signaling responses of these CaSR-expressing cells were assessed following stimulation with extracellular calcium (Ca^2+^_e_) using luciferase reporter assays for NFAT and SRE, which are downstream indicators of Ca^2+^_i_ mobilization and MAPK signaling, respectively (17). The NFAT and SRE responses of the CaSR-expressing cells increased in a concentration-dependent manner following stimulation with increasing Ca^2+^_e_ concentrations (Fig. 4A and 4B). However, cells expressing the variant (Pro448) CaSR protein or the FHH1-causing (Pro173) CaSR protein showed significantly reduced NFAT and SRE responses compared to WT cells (Fig. 4A and 4B). Indeed, cells expressing the Pro448 CaSR protein showed a rightward shift in the NFAT and SRE concentration-response curves with significant increases in the respective EC_50_ values when compared to WT CaSR (Fig. 4A and 4B), whereas cells expressing the Pro173 CaSR protein showed significant reductions in the maximal NFAT and SRE fold-change responses compared to WT CaSR (Fig. 4A and 4B). Thus, functional studies of the p.Ser448Pro CaSR variant demonstrate this to represent a loss-of-function CaSR mutation.

## Discussion

These findings, which have identified a novel p.Ser448Pro CaSR mutation in an FHH1 kindred, demonstrate that the infant of a mother with FHH1 can be hypercalcemic, normocalcemic, or hypocalcemic, depending on the inheritance of the maternal CaSR mutation. Thus, children harboring a maternally inherited loss-of-function CaSR mutation typically have an FHH1 phenotype with asymptomatic hypercalcemia during infancy, whereas children who have not inherited a loss-of-function CaSR mutation from a mother with FHH1 may either be normocalcemic or develop transient neonatal hypocalcemia, which can be symptomatic and cause seizures. However, not all unaffected infants of FHH1 mothers will develop symptomatic hypocalcemia, as highlighted by the unaffected normocalcemic son (individual III.1, Fig. 1A), who remained well during infancy. This suggests that additional factors, such as vitamin D deficiency, could have contributed to the marked hypocalcemia in the daughter (individual III.4, Fig. 1A).

Transient hypocalcemia affects around 1 in 50 term neonates (18), and can cause irritability and seizures (19). It is commonly associated with vitamin D deficiency and low or inappropriately normal PTH concentrations (18), both of which were features of this case. Reduced PTH secretion in the neonatal period can arise from delayed parathyroid gland maturation, hypomagnesemia (18), or as a consequence of maternal hypercalcemia, which suppresses fetal parathyroid activity (19). The major cause of maternal hypercalcemia is primary hyperparathyroidism, which affects approximately 1 out of 3000 women of reproductive age (20). In contrast, FHH1 has rarely been reported as a cause of hypercalcemia in pregnancy, and this condition may remain asymptomatic throughout gestation (21). However, it is possible that some mothers with FHH1 may develop more severe hypercalcemia in the third trimester because of increases in circulating PTH-related peptide concentrations (22), and therefore warrant serum calcium monitoring during the later stages of pregnancy. In addition, the present case indicates that maternal FHH1 poses a risk to the unaffected neonate because of suppression of the parathyroid gland function in utero. The risk of transient neonatal hypoparathyroidism is likely related to the degree of maternal hypercalcemia, and it is notable that the maternal serum calcium concentration in this case, and the 2 reported cases of FHH-associated neonatal hypocalcemia (7, 8), was substantially elevated at greater than 2.90 mmol/L (> 11.6 mg/dL). These findings indicate the importance of assessing maternal serum calcium as part of the evaluation of an infant with hypocalcemia, particularly because this may unmask the presence of FHH in the mother. Furthermore, serum calcium should be assessed at birth in the infant of a mother with FHH1 and also during the first 1 to 2 weeks after birth, and CaSR mutational analysis considered for the newborn offspring of FHH1 parents.

The p.Ser448Pro mutation identified in this FHH1 family is located within the CaSR ECD. This region of the CaSR binds a range of extracellular ligands, which include calcium, magnesium, and amino acids (13). However, our structural analysis showed that the mutated Ser448 residue is not located at one of the ECD ligand binding sites, but instead contributes to the ECD dimer interface, which is involved in agonist-mediated activation of the CaSR, and reported to be a hotspot for loss-of-function CaSR mutations (13, 17). Although the p.Ser448Pro mutation did not significantly alter the abundance of dimeric CaSR protein, our functional studies demonstrated the p.Ser448Pro mutation to cause a loss of function by impairing CaSR-mediated signaling via the Ca^2+^_i_ and MAPK pathways. The p.Ser448Pro mutation was associated with a greater than 30% increase in the EC_50_ value when compared to WT both in the in vitro NFAT and SRE assays (Fig. 4A and AB), and this degree of loss of function is similar to that reported for other FHH1-causing CaSR mutations (17).

In summary, our studies have a identified a novel loss-of-function CaSR mutation that caused asymptomatic hypercalcemia in a mother and her children who had inherited the mutation, but was also associated with transient neonatal hypocalcemic seizures in one of her children who had not inherited the CaSR mutation.

## Abbreviations

Ca2+e: extracellular calcium
Ca2+i: intracellular calcium
CaSR: calcium-sensing receptor
EC_50_: half-maximal effective concentration
ECD: extracellular domain
FHH1: familial hypocalciuric hypercalcemia type 1
MAPK: mitogen-activated protein kinase
NFAT: nuclear factor of activated T cells
PMCA1: plasma membrane calcium ATPase
PTH: parathyroid hormone
SRE: serum-response element
WT: wild-type

## Additional Information

***Disclosure Summary:*** The authors have nothing to disclose.

***Data Availability:*** All data generated or analyzed during this study are included in this published article or in the data repositories listed in References.
